# Nocardia Brain Abscess Mimicking Metastases in an Immunocompromised Patient

**DOI:** 10.7759/cureus.20248

**Published:** 2021-12-07

**Authors:** Caren Stuebe, Samantha Dayawansa, Jason H Huang, Frank S Harris

**Affiliations:** 1 Surgery, Texas A&M College of Medicine, Temple, USA; 2 Neurosurgery, Baylor Scott & White Health, Temple, USA

**Keywords:** case reports, lung neoplasms, intracranial mass, brain abscess, infections, nocardia

## Abstract

The differential for ring-enhancing lesions of the brain is extensive, with patient characteristics, particularly immunologic status, crucial to the clinical plan. In immunocompromised patients with a single ring-enhancing lesion, aspergillosis, toxoplasmosis, and nocardial infections are considered. In the case of multiple ring-enhancing lesions, metastases often supersede opportunistic infections on the differential. We present an unusual case of multiple nocardial brain abscesses mimicking metastases in an immunocompromised patient with a history of narcolepsy.

## Introduction

*Nocardia* is a gram-positive, aerobic, branching filamentous bacteria that usually presents as an opportunistic pulmonary infection in immunocompromised patients [1]. *Nocardia asteroides*, *N. brasiliensis*, *N. caviae*, and *N. cyriacigeorgica* are four species found in humans, with *N. asteroides* being the most common [2,3]. The lungs are the principal site of infection given that *Nocardia* primarily enters the human body through inhalation [4]. The most common extrapulmonary site for *Nocardia* is the central nervous system (CNS) with 1-2% of all brain abscesses being due to *Nocardia* intracranial infections [5,6]. Systemic nocardiosis usually occurs through hematogenous spread. A septic focus is the most common source and can result in single or multiple nocardial brain abscesses [6, 7]. Symptoms of nocardial CNS infection are typically gradual in onset and may include alterations in consciousness, seizures, headache, nausea, and vomiting. Rapid symptom progression may occur in the acute setting [5]. Though *Nocardia* is a rare cause of intracranial abscesses, nocardial brain abscesses carry a 31% mortality rate [6,8]. Thus, prompt diagnosis and treatment are crucial.

## Case presentation

A 67-year-old man with a history of narcolepsy and prior history of smoking was admitted to the ED following a motor vehicle accident (MVA). The patient presented with difficulty breathing secondary to rib fractures but denied pain, nausea, vomiting, changes in vision, numbness, weakness, and urinary incontinence. When questioned, the patient had no memory of the accident. The patient was compliant with his narcoleptic medications and, prior to this incident, had no accidents or unconscious spells. The patient thus attributed this accident and the associated memory lapse to “something new in his head.” The patient was followed by pulmonology for a current history of nonspecific interstitial pneumonia treated for the past eight months with prednisone. The patient was last seen six weeks prior to the MVA for interstitial pulmonary disease, nonspecific interstitial pneumonia, and wheezing that initially improved with steroids.

**Figure 1:**
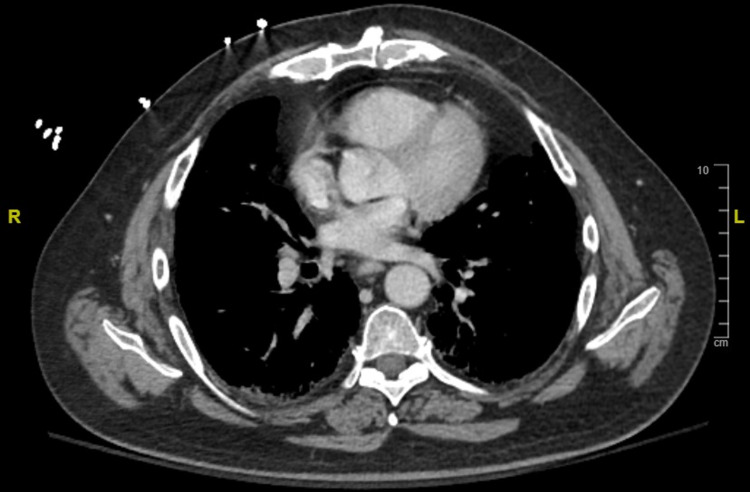
CT of the chest without contrast with no specific findings

**Figure 2:**
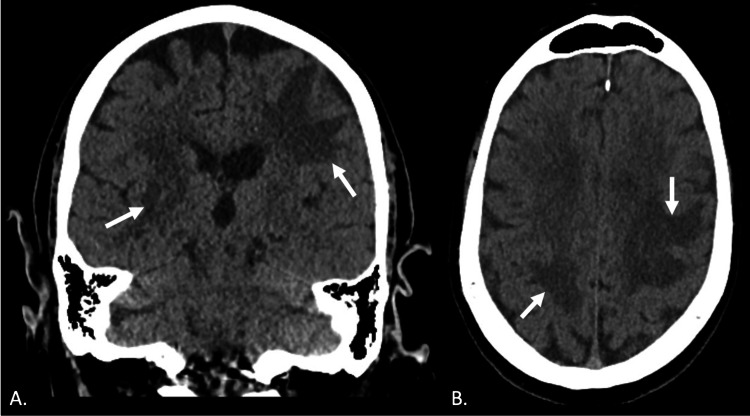
CT of the head without contrast: coronal (A) and horizontal (B) sections with arrows indicating areas of vasogenic edema

**Figure 3:**
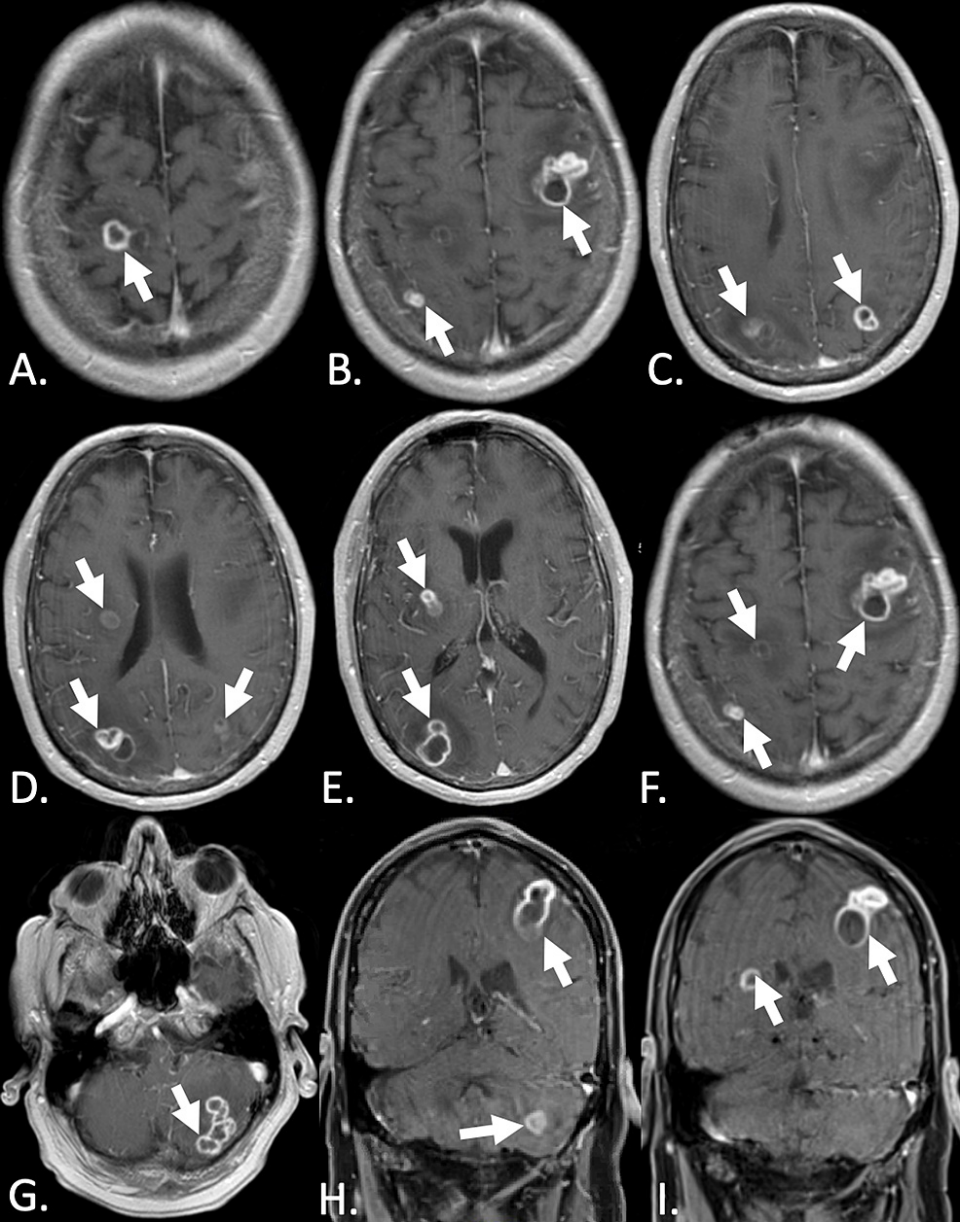
MRI of the brain: horizontal (A-G) and coronal (H&I) MRI sections with arrows indicating multiple ring-enhancing lesions

Initial imaging work-up included CT of the chest (Figure 1), CT of the head without contrast (Figure 2), and MRI of the brain (Figure 3). CT chest was unremarkable (Figure 1). CT head without contrast was significant for vasogenic edema (Figure 2). MRI brain revealed multiple ring-enhancing lesions including a significant left frontal lesion (Figure 3).

**Figure 4:**
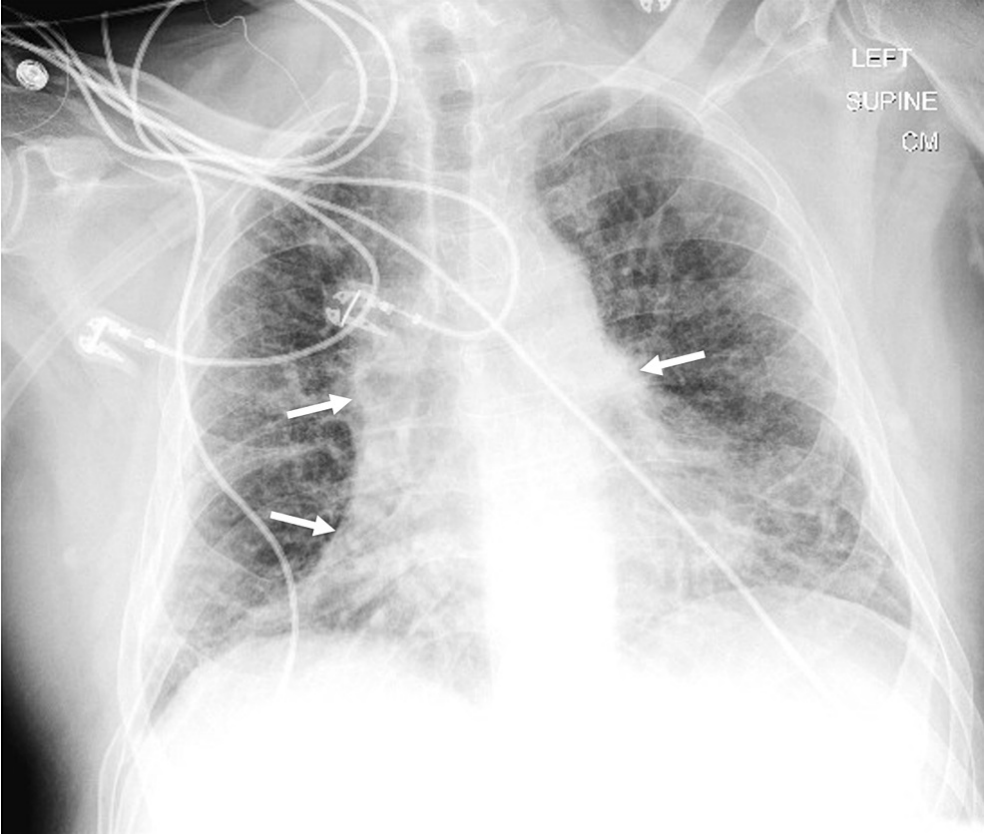
X-ray of the chest Arrows showing bilateral reticulonodular infiltrates and lobar consolidation indicative of pulmonary nocardiosis.

The initial diagnosis was multiple metastases given the imaging findings and diffuse distribution of lesions. However, the patient’s immunocompromised status widened the differential. Stereotactic biopsy and pathologic evaluation of the left frontal lesion identified a cerebral abscess with focal granulation reaction containing fragments of brain tissue with inflammatory infiltration including neutrophils, lymphocytes, and plasma cells. Periodic-acid Schiff stain for fungal microorganisms was negative. Immunostaining for keratin was negative. The culture of the abscess grew *Nocardia*. Chest x-ray (Figure 4) was characteristic of chronic *Nocardia*, and thus supported the culture finding. The patient was diagnosed with a left frontal nocardial abscess and placed on a six-week combination therapy of trimethoprim/sulfamethoxazole and ceftriaxone followed by amoxicillin/clavulanic acid and minocycline. The patient is tolerating the amoxicillin/clavulanic acid and minocycline combination therapy well, with planned repeat MRI brain in a few months and continued combination therapy for one year. 

## Discussion

*Nocardia* is an opportunistic pathogen most common in male patients between 20 and 60 years of age [5]. Compared to females, males have a higher risk for infection with a recently reported incidence of 3:1 [9]. Uttamachandi et al. and Palmer et al. also reported a higher incidence in men, at 4:1 and 2:1, respectively [10,11]. The higher incidence in males is most likely due to exposure frequency, especially for males with outdoor occupations, rather than differences in susceptibility between males and females. This organism most often presents clinically in the pulmonary system through extrapulmonary nocardiosis, specifically to the CNS, which is fairly common. CNS nocardiosis can present with single or multiple brain lesions [6,7]. In immunocompromised patients especially, metastatic disease and serious clinical complications can result. However, the ubiquity of *Nocardia* allows that both immunocompromised and immunocompetent individuals can be affected [6]. 

In the case of our patient, his pre-existing pulmonary disease and immunocompromised status secondary to an eight-month corticosteroid course predisposed him to the contraction of *Nocardia*. It is possible that the MVA that brought the patient into the hospital was secondary to focal neurologic symptoms, including alterations in consciousness and seizures, from the nocardial brain abscesses. The absence of similar episodes and stable symptomology of the patient while on his narcoleptic medications points to a nocardial-brain abscess, rather than a narcoleptic-related cause for the accident-associated amnesia. The onset of neurological symptoms is a hallmark of nocardial CNS involvement. Mamelak et al. reported the most common findings in CNS nocardiosis patients were focal neurological deficits (42%), epileptic seizures (30%), and non-focal deficits (27%) [12].

In the case presented by Karan et al., the patient first presented with a lung lesion, refused follow-up, and two months later developed neurological symptoms including generalized seizures [6]. The presence of the prior lung lesion prompted suspicion of metastasis. Like in our patient, pathologic evaluation of the specimen revealed *Nocardia*. Unlike our patient, the lung, rather than brain lesion, was identified first, and refusal of further work-up allowed progression to neurological symptoms. In addition, Karan et al. did not report a patient history consistent with an immunocompromised state [6].

Similarly, Menkü et al. reported two immunocompetent patients who presented with initial neurological symptoms later identified via CT imaging and pathological examination to stem from Nocardia brain lesions [4]. Several other reports of CNS nocardia in immunocompetent patients have been cited in the literature [13-16]. While the mortality rate of immunocompromised patients with CNS nocardia is 55%, Mamelak et al. report a 20% mortality rate in immunocompetent patients [12].

In addition to the immunocompetent status of both patients, the Menkü et al. cases are interesting in that neither patient presented with pulmonary findings. Thus, while the pulmonary infection was found in our patient and others in the literature, there are cases reported by others, including by Menkü et al., with an absence of pulmonary infection [4,15,17]. The presence of a primary *Nocardia* intracranial infection without associated pulmonary disease is unusual, as is the presence of CNS nocardia in immunocompetent patients. Both of these instances emphasize the utility of a wide differential diagnosis in the work-up of ring-enhancing brain lesions, even in immunocompetent patients.

Clinical findings, most specifically neurological findings, prompt imaging investigation in Nocardia patients. CT is usually the initial method for screening. If performed with IV contrast, the CT reveals singular or multiple ring-enhancing lesions with surrounding vasogenic edema. Our patient received a non-contrast CT and thus only presented with vasogenic edema. At this stage, an MRI of the brain is usually ordered for greater sensitivity. Still, a distinction between a brain abscess and another brain lesion can be difficult with MRI alone. Magnetic resonance proton spectroscopy (MRS) and diffusion-weighted imaging (DWI) are sometimes employed to better differentiate the lesion [18-20]. However, subsequent work-up with pathologic examination, in addition to imaging and clinical findings, is required for definitive diagnosis. In the case of our patient, the MRI brain was initially thought to be neoplastic, in line with the initial diagnosis of several other similar cases in the literature [4,13,15,16]. Biopsy with a pathological examination, as in our patient, led to the diagnosis of CNS nocardiosis.

Accurate diagnosis of CNS nocardiosis is essential for informed treatment. While corticosteroid therapy contributes to the reduction of cerebral inflammation and improvement of clinical symptoms in CNS tumor patients, it worsens prognosis in CNS nocardia patients. If a nocardial brain abscess is misdiagnosed as a tumor and corticosteroid therapy is begun, the abscess can rapidly progress and result in the expedited clinical deterioration of the patient [16]. Once identified as a nocardial brain abscess, antimicrobial therapy and surgical excision are the most common treatment options. While there are no prospective randomized trials on the most effective *Nocardia* antimicrobial treatment, the proposed regimen involves combination treatment with two to three agents, with one agent usually a sulfonamide [4]. Aspiration and complete resection are the proposed surgical treatments of *Nocardia*. Lee et al.found aspiration alone is sufficient in the majority of patients [21]. When mortality is evaluated, Mamelak et al.found a 24% mortality rate in those requiring craniotomy and excision whereas the mortality rate of aspiration and drainage was two times as high at 50% [9]. Our patient received a left frontal brain biopsy with a diagnosis of nocardial brain abscess, was started on combination therapy of trimethoprim/sulfamethoxazole and ceftriaxone for six weeks, and then was transitioned to amoxicillin/clavulanic acid and minocycline planned for one year.

## Conclusions

Our case is unusual in the presence of multiple ring-enhancing lesions on cranial imaging of an immunocompromised patient. While a single ring-enhancing lesion, particularly in an immunocompromised patient, often raises suspicion for a brain abscess, the presence of multiple ring-enhancing lesions often points to a neoplastic process. The mimicry between abscesses and neoplasms (including metastases, lymphoma, and high-grade astrocytoma) is a continued diagnostic challenge that warrants work-up regardless of immunologic status. 
